# The effect of long-acting injectable antipsychotics on QRISK3 in patients with bipolar disorder and schizophrenia

**DOI:** 10.1192/j.eurpsy.2023.624

**Published:** 2023-07-19

**Authors:** V. Erbasan, F. Ekici, E. F. Uçak, M. Aydın, K. Altınbaş

**Affiliations:** ^1^Psychiatry, İzmir Tepecik Training and Research Hospital Clinics, İzmir; ^2^Psychiatry, Konya City Hospital; ^3^Psychiatry, Selçuk University Medical Faculty, Konya, Türkiye

## Abstract

**Introduction:**

Psychiatric diseases are often accompanied by medical comorbidities, and cardiovascular system disease is one of the most important causes of death in bipolar disorder (BD) and schizophrenia (SCH) [1]. The QRISK3 algorithm calculatesa person’s risk of developing cardiovascular disease over the next ten years and it has been used in psychiatric research in recent years [2].

**Objectives:**

In this study, we aimed to evaluate the relationship between cardiovascular risks (by using QRISK3 algorithm) and treatment options in patients of BD and SCH.

**Methods:**

Thirty-six patients with bipolar disorder and thirty-two patients with schizophrenia recruited between the years 2016-2022. All of the patients were treated with long-acting injectable (LAI) antipsychotics. Sociodemographic information, clinical features and medical treatment agents of individuals were recorded. The risk of development of cardiac disease of the patients were evaluated by the QRISK3 algorithm. Descriptive and regression statistical analysis was performed with Statistical Package for the Social Sciences (SPSS).

**Results:**

More than half of the patients were male (n= 39, 57.4%) and single (n= 40, 58.8%). The mean duration of education was 9.2 ±3.5 years and the mean age was 39.7 ±11.9 years of the patients. Nearly half of the patients had a diagnosis of BD (n= 36, 52.9%) while others had SCH (n:32, 47.1%). Most of the patients were treated with LAI of aripiprazole (n= 36, 59.2%) and the rest of them were treated with LAI of paliperidone (n= 32, 40.8%). When demographic and clinical data of BD and SCH patients were compared, there was no significant difference in terms of age. However, SCH patients had a longer disease duration (t= 2.56, p= 0.013) and more hospitalization (t= 3.35, p= 0,002) than BD patients. Our findings showed positive correlations between age (r= 0.74, p<0.01), duration of disease (r= 0.57, p< 0.01), and duration of LAI use (r= 0.55, p< 0.01) with QRISK. A regression analysis to examine related factors associated with QRISK found that having a diagnosis of schizophrenia (β= 0.27, t= 2.58; p= 0.01) and a long disease duration (β= 0.67, t= 6.50, p< 0.01) were associated with the development of cardiovascular disease. However, there was no relationship between LAIs (paliperidone vs aripiprazole) used in the treatment and QRISK.

**Conclusions:**

The significant correlation found between duration of disease and QRISK -that indicate the risk of development of cardiac disease- confirming the progressive nature of the BD and SCH. Moreover, the significant correlation between SCH and QRISK suggests that SCH is a chronic and severe disease that causes more cardiac dysfunction than BD. However, we could not find a significant difference between LAIs in terms of QRISK, these results can be changed by evaluating other variables such as comorbidities, other medical treatments and physical activities in future research.

**Disclosure of Interest:**

None Declared

